# Mutually Exclusive Expression of Virulence Genes by Malaria Parasites Is Regulated Independently of Antigen Production

**DOI:** 10.1371/journal.ppat.0020022

**Published:** 2006-03-03

**Authors:** Ron Dzikowski, Matthias Frank, Kirk Deitsch

**Affiliations:** 1 Department of Microbiology and Immunology, Weill Medical College of Cornell University, New York, New York, United States of America; 2 Division of International Health and Infectious Diseases, Weill Medical College of Cornell University, New York, New York, United States of America; Northwestern University Medical School, United States

## Abstract

The primary virulence determinant of Plasmodium falciparum malaria parasite–infected cells is a family of heterogeneous surface receptors collectively referred to as PfEMP1. These proteins are encoded by a large, polymorphic gene family called *var*. The family contains approximately 60 individual genes, which are subject to strict, mutually exclusive expression, with the single expressed *var* gene determining the antigenic, cytoadherent, and virulence phenotype of the infected cell. The mutually exclusive expression pattern of *var* genes is imperative for the parasite's ability to evade the host's immune response and is similar to the process of “allelic exclusion” described for mammalian Ig and odorant receptor genes. In mammalian systems, mutually exclusive expression is ensured by negative feedback inhibition mediated by production of a functional protein. To investigate how expression of the *var* gene family is regulated, we have created transgenic lines of parasites in which expression of individual *var* loci can be manipulated. Here we show that no such negative feedback system exists in P. falciparum and that this process is dependent solely on the transcriptional regulatory elements immediately adjacent to each gene. Transgenic parasites that are selected to express a *var* gene in which the PfEMP1 coding region has been replaced by a drug-selectable marker silence all other *var* genes in the genome, thus effectively knocking out all PfEMP1 expression and indicating that the modified gene is still recognized as a member of the *var* gene family. Mutually exclusive expression in P. falciparum is therefore regulated exclusively at the level of transcription, and a functional PfEMP1 protein is not necessary for viability or for proper gene regulation in cultured parasites.

## Synopsis

Mutually exclusive gene expression refers to the ability of an organism to select one member of a large, multicopy gene family for expression while simultaneously silencing all other members of the family. Human malaria parasites utilize this process in regulating the expression of the major antigenic and virulence-determining proteins encoded by a multicopy gene family called *var*. In any given parasite, only a single *var* gene is expressed at a time, while all other members of the family are transcriptionally silenced. The mechanism that regulates this tightly controlled process and coordinates switches in gene expression is largely unknown. Here Dzikowski and colleagues show that this process is regulated entirely at the level of transcription, and that protein production and chromosomal context of the genes are not involved. In addition, they identify the DNA elements required for a *var* gene promoter to be recognized and co-regulated along with the rest of the family. This knowledge has enabled the authors to create transgenic parasites in which they can manipulate expression of the entire *var* gene family through selection for expression of specific, modified *var* genes, thus knocking out expression of the main virulence factor of malaria.

## Introduction

Plasmodium falciparum is the protozoan parasite responsible for the deadliest form of human malaria, causing more than one million deaths a year [1]. The most prominent virulent surface antigen expressed by P. falciparum is the protein PfEMP1 (P. falciparum erythrocytic membrane protein 1) encoded by the multicopy *var* gene family [2–4]. This protein is thought to be the primary antigenic molecule on the infected cell surface as well as the major determinant of the cell's cytoadherent and virulence properties. Over the course of an infection, parasites regularly switch which PfEMP1 is expressed, thus avoiding the antibody response specific to previously expressed forms of PfEMP1 and mediating the process of antigenic variation [5]. This process is regulated at the level of *var* gene transcription and depends on the fact that only one gene is expressed at a time in a single parasite [6,7]. The P. falciparum genome contains approximately 60 *var* genes [8]; however, frequent recombinations, deletions, and gene conversions create an endless *var* repertoire for antigenic variation. The processes of mutually exclusive *var* gene expression, rapid switching of the expressed gene, and the ability to generate a virtually limitless collection of new *var* genes is thought to be responsible for the fact that complete immunity to malaria infection is difficult or impossible to achieve.

There are many examples of mutually exclusive gene expression described in several organisms, including dosage compensation [9] and imprinting in mammals [10] and VSG expression in African trypanosomes [11]. While the molecular mechanisms that regulate this process are not completely understood in any eukaryotic system, many advances have been made recently with regard to the regulation of the mammalian Ig heavy-chain genes expressed in B cells as well as the odorant receptor gene family expressed in olfactory sensory neurons [12,13]. In both of these examples, the genes encode cell surface receptors that are expressed in a mutually exclusive, mono-allelic manner, leading to the “one cell–one receptor” paradigm. This phenomenon is frequently referred to as “allelic exclusion,” and the ultimate decision as to which allele will be expressed in an individual cell has been shown to depend on negative feedback at the level of protein expression. Replacement of the receptor coding region with that of a reporter gene, or disruption of the open reading frame lead to activation of an additional allele, thus confirming the model that mono-allelic gene expression is ultimately regulated via the production of a functional protein on the cell surface. In the case of the Ig heavy-chain genes, the inhibitory feedback signal has been shown to require the spleen tyrosine kinase [14], leading to silencing of the alternative locus through modifications in chromatin structure.

Regulation of expression of the *var* gene family has many similarities to the systems described above. *var* genes are likewise expressed in a mutually exclusive manner and encode cell surface receptors. In addition, epigenetic alterations in chromatin structure appear to be important for maintaining “off” genes in a transcriptionally silent state [15,16]. However, it is not known whether a negative feedback mechanism exists requiring production of a functional PfEMP1 protein from one *var* gene to repress expression of other members of the gene family. Previously, Gannoun-Zaki et al. [17] had shown that a *var* promoter driving expression of a drug-selectable marker rather than PfEMP1 is not recognized by the mechanism that controls *var* allelic exclusion. We have subsequently confirmed this result, suggesting the possibility that a negative feedback mechanism does exist. However, the *var* promoter in this construct is constitutively active because it has been separated from a silencing element found in *var* introns [17]. Therefore it is unclear if this promoter remains “uncounted” because it fails to produce a functional protein or rather if its separation from potential regulatory elements in the intron and elsewhere in the regulatory regions surrounding the gene has simply made it unrecognized by the process that controls allelic exclusion.

To begin to address the question of allelic exclusion in *P. falciparum,* we created transgenic parasite lines where we could select for transcriptional activation of specific *var* promoters. In these genetically modified parasites, recombinant *var* promoters drive expression of a drug-selectable marker rather than the surface protein PfEMP1. These promoters are initially silenced, implying that they are properly regulated. However, selection for activation results in the silencing of all other members of the *var* gene family, indicating that these promoters are indeed “counted” by the mechanism that regulates mutually exclusive *var* gene expression. These results indicate that mono-allelic expression of *var* genes depends solely on noncoding elements at each *var* gene and is independent of production of a functional PfEMP1 protein.

## Results

### Integration of a Selectable Marker into Internal and Subtelomeric Chromosomal *var* Loci

To acquire accurate data regarding transcriptional regulation, it is preferable to study a *var* promoter within its chromosomal context that can be both transcriptionally silenced and activated. For this purpose we utilized constructs designed to integrate directly into a *var* locus in such a way as to preserve any surrounding elements that might regulate gene expression. These constructs carry two selectable markers: the Blaticidin S deaminase *(bsd)* gene behind a *var* upstream region and the human dihydrofolate reductase *(hdhfr)* gene driven by the promoter activity of a *var* intron. The presence of a *var* intron on these plasmids ensures that the upstream *var* promoter driving *bsd* expression can be silenced [17]. The *var* upstream regions included in the constructs were truncated to include only sequences downstream of the transcription start site, thus preventing expression of the *bsd* gene without homologous integration into the correct chromosomal *var* upstream region. This design enables rapid positive selection for integration into the desired locus as described by Wang et al. [18]. The constructs, cloning strategy, and expression patterns are shown in Figures 1–5. *var* loci are found in both subtelomeric and internal regions of the chromosomes, and it has been speculated that chromosomal location might influence gene expression patterns [19]. Therefore we targeted our constructs to both internal and subtelomeric *var* loci by using UpsB (typical subtelomeric promoter) and UpsC (typical internal promoter) [19,20] upstream regions in the plasmids pV_b_BB/IDH (Figures 1A and 4A) and pV_c_BB/IDH (Figure 5A), respectively. A recently cloned population of P. falciparum parasites (NF54/C3) predominantly expressing *var* PFD1005c (Figure 3, top panel) was transfected as previously described [21,22], selected using pyrimethamine until resistant lines stably containing episomes were established and then grown in the presence of blasticidin to select for integration at the targeted *var* loci. At this point episomes could no longer be detected by “plasmid rescue.” Limiting dilution was then used to clone distinct genetic lines from the established blasticidin resistant culture. The sites of integration were determined by PCR (polymerase chain reaction) and confirmed by sequencing across integration sites and Southern blotting. To monitor *var* gene transcription levels and to examine the expression state of the rest of the *var* gene family, we used quantitative real-time RT-PCR (Q-RT-PCR) analysis applying the primer set developed by Salanti et al. [23]. Unlike simple hybridization techniques, this approach enables simultaneous monitoring of the transcription levels of each individual gene within the entire *var* family of the P. falciparum NF54 line.

**Figure 1:**
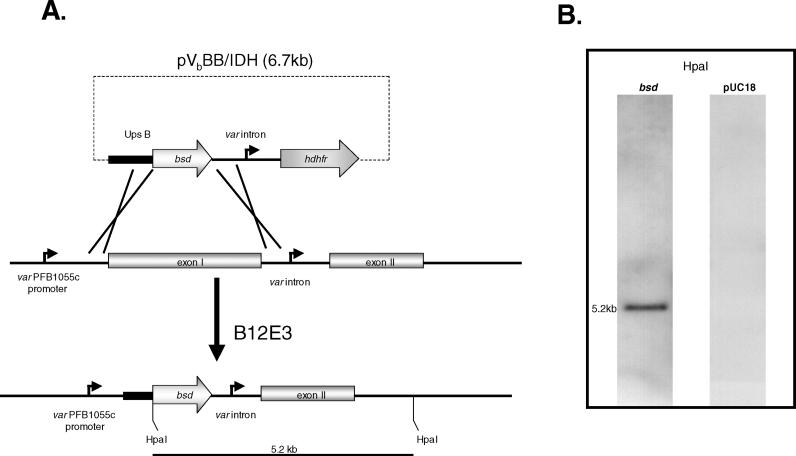
Replacement of PfEMP1 Exon I with the Selectable Marker *bsd* (A) Schematic diagram showing integration of the *bsd* cassette from the construct pV_b_BB/IDH into the NF54 *var* PFB1055c locus through double crossover recombination. (B) Southern analysis was performed using HpaI digested DNA and probed for *bsd* and pUC18. The size of the DNA fragment is shown on the left. The absence of a 6.7-kb band when using a *bsd* probe and the absence of hybridization with plasmid backbone are indicative of a double crossover recombination event, resulting in replacement of exon I with the *bsd* cassette. This arrangement was confirmed by additional Southern blots and by sequencing across the sites of integration.

**Figure 2:**
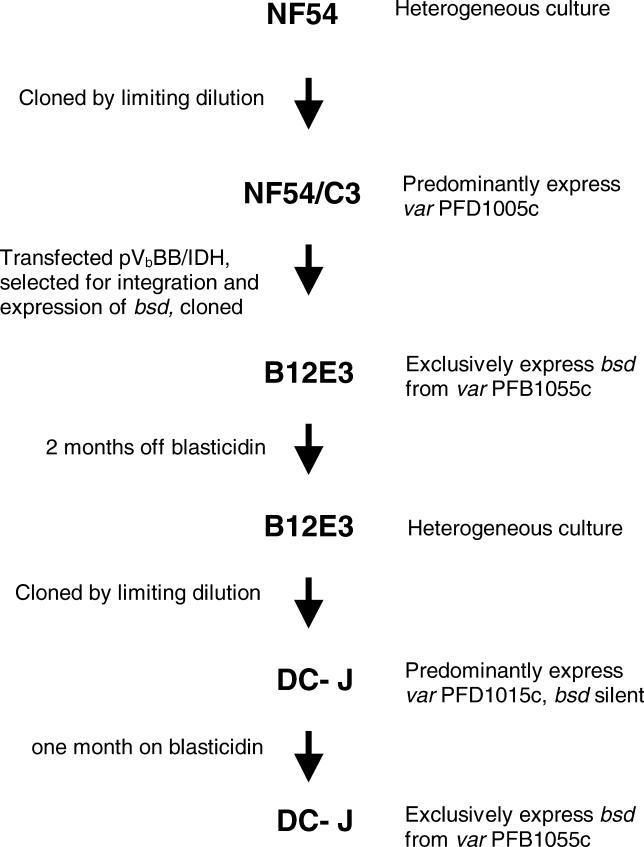
Outline of Experimental Design NF54 parasites were cloned by limiting dilution to create a clonal population (NF54/C3) that predominantly expressed PFD1005c. These parasites were transfected with the plasmid pV_b_BB/IDH and the clone B12E3 containing a double crossover integration was isolated. After blasticidin selection, B12E3 exclusively expressed *bsd,* and all other *var* genes were transcriptionally silent. Drug pressure was then removed for two months during which time the *var* gene expression pattern became heterogenous. This heterogeneous B12E3 population was then re-cloned and clone DC-J isolated. DC-J predominantly expressed the *var* gene PFD1015c and had silenced the *bsd* gene. Growth of DC-J back under blasticidin pressure however results in reactivation of *bsd* expression and silencing of the rest of the *var* gene family, thus demonstrating the reversibility of the phenomenon.

**Figure 3:**
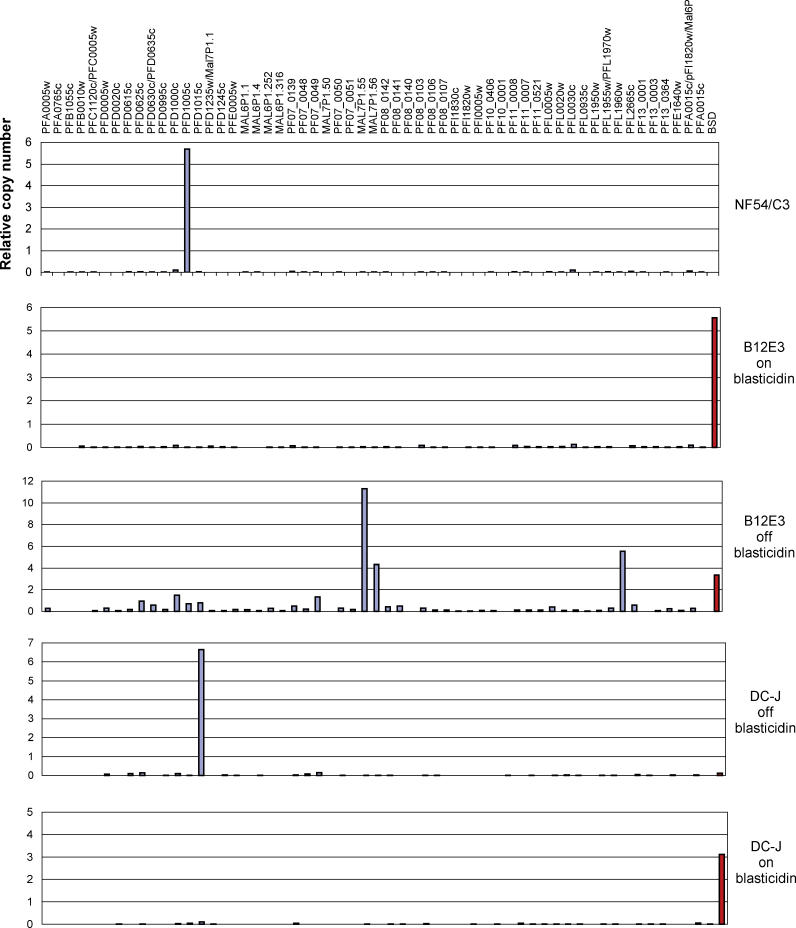
Analysis of Levels of Transcription of the Entire *var* Family All values are presented as relative copy number to the housekeeping gene seryl-tRNA synthetase (PF07_0073). Top panel: NF54 parasites were cloned by limiting dilution, and *var* gene expression was measured by Q-RT-PCR as soon as the culture reached the required parasitemia, approximately 6 wk after plating. The clone NF54/C3, which was used to generate all transgenic lines, was predominantly expressing *var* PFD1005c (located on Chromosome 4 internal cluster) while expression of the rest of the *var* family was virtually undetectable. Second panel: The recombinant line B12E3 growing under blasticidin pressure only transcribed *bsd* (red), while transcription levels of the rest of the *var* family was close to zero (blue). Central panel: Expression of additional *var* genes was easily observed after the parasites were grown for 10 wk without drug pressure. At this point the culture is transcriptionally heterogeneous and *bsd* is no longer the dominant gene. Five genes were expressed at levels equal to or greater than the control (copy number = 1). Fourth panel: The transcription pattern of clone DC-J that was re-cloned from this culture represents a population that had switched away from *bsd* and now predominantly expresses *var* PFD1015c. Applying blasticidin to DC-J resulted in the selection of parasites that had switched back to exclusively expressing *bsd* (bottom panel).

**Figure 4:**
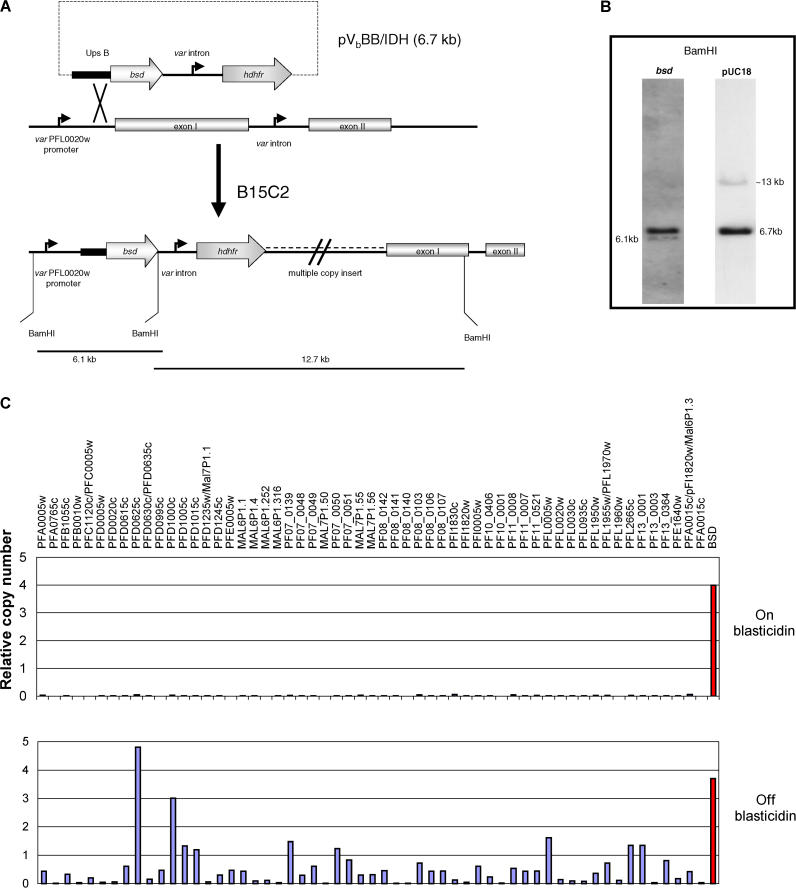
Integration of the *bsd* Expression Cassette into a Telomeric *var* Locus (A) Schematic diagram showing integration of the construct pV_b_BB/IDH into the *var* PFL0020w locus through a single homologous recombination event within the *var* upstream region. (B) Southern analysis was performed using BamHI-digested gDNA and hybridized with probes specific to *bsd* and pUC18. The linearized plasmid within the multiple copy concatameric insertion appears as a high intensity 6.7 kb band with both probes. The 6.1-kb *bsd* band and the ~13-kb pUC18 band correspond to fragments flanking the site of integration. This arrangement was confirmed by additional Southern blots and by sequencing across the sites of integration. (C) Analysis of the level of transcription from each *var* gene in the genome shows that selection for *bsd* expression results in silencing of the entire gene family. The only “on” gene in parasites growing under drug pressure (top panel) is the locus expressing the *bsd* cassette. Analysis performed 10 wk after drug removal demonstrated that the culture has become transcriptionally heterogenous (bottom panel) with the majority of the genes upregulated, of which nine are expressed at levels greater than the control (relative copy number > 1).

**Figure 5:**
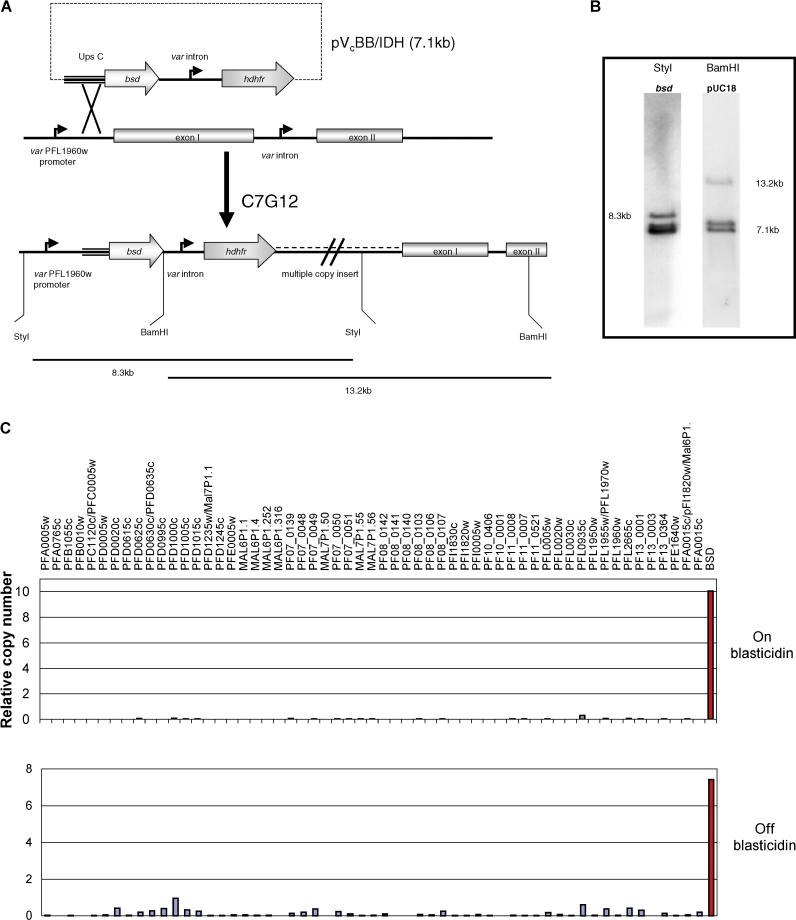
Integration of a *bsd* Expression Cassette into an Internal Chromosomal *var* Locus (A) Schematic diagram showing integration of the construct pV_c_BB/IDH into the *var* PFL1960w locus through a single homologous recombination event within the *var* upstream region. (B) Southern analysis was performed using BamHI and StyI digested gDNA hybridized with probes to *bsd* and pUC18. The 8.3-kb *bsd* band and the 13.2-kb pUC18 band correspond to the fragments flanking the integrated concatamer. The doublet at ~7.1 kb corresponds to fragments from within the integrated concatamer. (C) Analysis of the level of transcription of each *var* gene within the parasite genome shows that selection with blasticidin results in silencing of all members of the *var* gene family. The only *var* locus expressed by parasites grown under drug pressure (top panel) was the gene that contained the integrated *bsd* expressing plasmid. 10 wk after drug removal, the culture became transcriptionally heterogenous (bottom panel).

Several different recombinant lines were analyzed. In one line (B12E3), a single copy double crossover integration into the subtelomeric *var* gene PFB1055c (on Chromosome 2) was detected. This integration was the result of one crossover in the *var* upstream region and a second within the intron sequence upstream of the *hdhfr* gene and resulted in exon I being replaced by the *bsd* gene (Figure 1A). Several additional recombinant lines were also isolated and found to contain integration of the constructs into either the subtelomeric *var* gene PFL0020W (clone B15C2, promoter type UpsB, on Chromosome 12, Figure 4A) or the internal *var* gene PFL1960w (clone C7G12, promoter type UpsC, Chromosome 12, Figure 5A). Analysis of the integration events using quantitative, real-time PCR and Southern blots of genomic DNA indicated that each parasite contained a concatamer of 5–10 copies integrated at the targeted locus. Because the plasmid construct only contained a nonfunctional, truncated *var* promoter, the only *bsd* copy capable of being expressed is that which is immediately downstream of the endogenous *var* promoter at the site of integration. Thus, in each case the targeted endogenous *var* upstream region was now driving the blasticidin resistant gene rather than the PfEMP1 coding region.

### Expression of a Transgene from a *var* Locus Reversibly Silences the Entire *var* Gene Family

In the B12E3 transgenic line, the double crossover event resulted in the replacement of exon I with the *bsd* coding region, but left intact the remainder of the locus, including the entire upstream region, the intron, exon II and the downstream region (Figure 1). This integration event therefore contains little disruption of any regulating elements that may surround the coding region of the gene. To evaluate how this modified *var* gene is regulated, and to investigate how expression of this gene affects the rest of the *var* gene family, the cloning strategy shown in Figure 2 was completed. Expression levels of each *var* gene in the genome as well as the inserted *bsd* gene were monitored at each step in the selection and cloning process and are shown in Figure 3.

Transcription-level quantification of ring-stage parasites showed that in B12E3 parasites kept under *bsd* pressure the only active *var* promoter in the genome was the one driving *bsd,* while the rest of the *var* family was silent (Figure 3, second panel). Removal of the drug resulted in gradual activation of other *var* genes over time, indicating that in the absence of drug selection, *var* gene expression may be switching away from the modified locus, resulting in the activation other *var* genes. Three weeks after drug removal, an increase in the level of transcription was detected in a few *var* genes, although no *var* transcript reached the level of the *bsd* gene. However, 10 wk after drug removal, *Mal7P1.55* was then the most highly transcribed *var* gene, transcripts from two other genes reached the level of *bsd,* and a few others reached the level of the housekeeping gene (seryl-tRNA synthetase, PF07_0073) (Figure 3, third panel). To determine if the activation of other *var* genes is the result of true expression switching or is instead due to deletion of the transgene, we re-cloned this transcriptionally heterogeneous population without drug pressure and evaluated *var* gene transcription in several of the resulting individual clones. In eight isolated clones, three demonstrate true epigenetic switching, four did not switch and continued exclusively expressing *bsd,* and one had deleted the *bsd* transgene. As shown in Figure 3 (fourth panel), in the DC-J clone the *bsd* gene switched to a silent state while *var* PFD1015c was actively transcribed, thus demonstrating a switch in *var* gene expression. Quantification of gene copy number by gDNA realtime PCR confirmed that the *bsd* gene was still present in the genome as a single copy gene in these parasites, but was now transcriptionally silent. Interestingly, the gene that became activated is located in a Chromosome 4 internal cluster adjacent to the gene that was active in the originally transfected NF54/C3 clonal population. To determine if the switch in expression is reversible, we put the DC-J line back under blasticidin pressure and were able to reselect a culture exclusively transcribing *bsd* (Figure 3, bottom panel). These results demonstrate that a *var* locus expressing an exogenous protein is recognized as an “on” *var* gene by the mechanism that controls mutually exclusive *var* gene expression, and that silencing and activation of such a gene is a reversible, epigenetic process.

### Active *var* Promoters Separated from PfEMP1 Coding Regions by Multicopy Inserts Induce Silencing of the *var* Gene Family

In the double crossover line B12E3, the coding region of exon I was replaced with the drug-selectable marker while keeping the gene structure intact. We asked whether the multicopy inserts in the subtelomeric *var* gene PFL0020W and the internal *var* gene PFL1960w that separate the active *var* promoter from the PfEMP1 coding region would still be recognized by the *var* allelic exclusion mechanism. In these clones the endogenous *var* promoters at the site of integration were separated from the PfEMP1 coding region by multiple copies of the plasmid; however, they are still paired with a *var* intron and any regulatory elements it contains (Figures 4A and 5A). Q-RT-PCR analysis of expression of the *var* family in clones containing either the subtelomeric or internal integration events grown under blasticidin pressure showed that only the *bsd* gene was transcribed at the level of an active *var* gene while the rest of the family was silent. Similar to the clone containing the double crossover event, removal of drug resulted in the gradual increase of transcription of other *var* genes in the population over time (Figures 4C and 5C).

To confirm that *var* gene expression could in fact be knocked out in the recombinant parasite lines, PfEMP1 protein expression was monitored by Western blot using polyclonal antisera raised against the conserved cytoplasmic domain of PfEMP1. These blots confirmed that while under blasticidin selection pressure, PfEMP1 expression could not be detected in the recombinant parasite lines, thus verifying the RNA expression data indicating that all other *var* genes in the parasite genome are silent (Figure 6). Taken together, the transcription and protein expression data indicate that noncoding regulatory elements at each *var* locus are important for recognition by the allelic exclusion mechanism, while production of a functional PfEMP1 protein is not.

**Figure 6:**
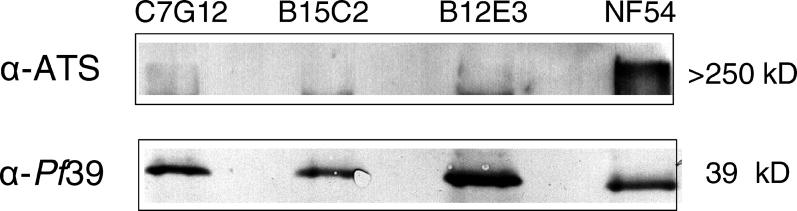
PfEMP1 Is Not Expressed in the Knock-Out Transgenic Lines Western blot analysis of whole cell extracts isolated from NF54 and the three transgenic lines B12E3, B15C2, and C7G12 growing under blasticidin pressure. Extract were probed with antibodies to either the conserved C terminus of PfEMP1 (α-ATS) or to the ER protein *Pf*39 (α *Pf*39). α-ATS signal appears only in NF45 parasites and not in any of the transgenic lines.

### Exclusive Expression Is Independent of Chromosomal Context

Within the genome of *P. falciparum, var* genes are found in specific chromosomal locations, either within subtelomeric regions or clustered in tandem arrays in the internal areas of the chromosomes. It has been proposed that the location and chromosomal context surrounding *var* genes may contribute to expression patterns or to recombination between genes [19,20]; however, the role that chromosomal context plays in mutually exclusive expression has not been directly considered. To address whether location within a chromosome is necessary for the “on” *var* promoter to be counted by the mechanism controlling mutually exclusive expression of the *var* family, we transfected the construct pVBB/IDH into the same NF54/C3 clonal population. This construct is similar to the constructs that were integrated into the chromosome with the exception that it includes a full-length *var* upstream region (UpsC) including the start site of transcription, thus allowing selection of parasites that have activated an episomal *var* promoter without integration into the genome. Parasites carrying stable episomes were isolated as described above using pyrimethamine selection for expression of the *hdhfr* gene driven by the promoter activity of the *var* intron. After verifying that the transformed parasites carried an intact pVBB/IDH, they were placed under blasticidin pressure to select for activation of the episomal *var* promoter and analysis of expression of the entire *var* family was performed. The active episomal *var* promoter was counted by the mechanism regulating exclusive expression and effectively silenced the rest of the endogenous *var* repertoire (Figure 7). This indicates that recognition of a *var* promoter and coordinated expression of the *var* gene family depends only on the noncoding elements at each *var* locus and is independent of chromosomal context. Further, these experiments demonstrate that all of the DNA elements necessary for recognition of a *var* promoter by the mechanism that controls mutually exclusive expression are included within the transfected construct.

**Figure 7:**
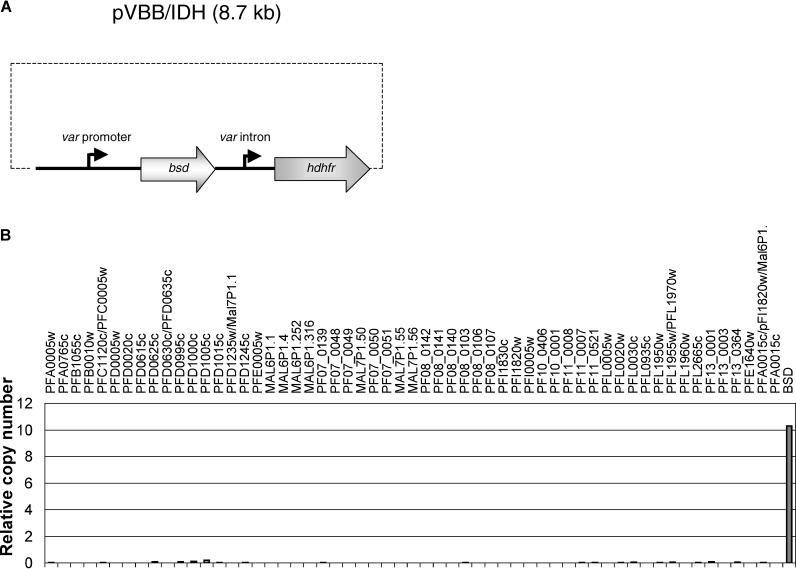
An Active *var* Promoter on an Episome Is Exclusively Expressed NF54/C3 parasites stably carrying the episome pVBB/IDH were grown under blasticidin pressure. (A) Plasmid map of pVBB/IDH. (B) Transcription levels were then measured by Q-RT-PCR and indicated that the parasites exclusively express *bsd* while all endogenous *var* genes are silent.

## Discussion

For the first time, expression of the entire *var* gene family can be knocked out in *P. falciparum* in a reversible way. We have created transgenic parasites where by using a drug-selectable marker we can now turn “on” different subtelomeric and internal *var* promoters and thus shut “off” transcription of the rest of the *var* family. This study shows that the expression of a transgene from *var* locus does not interrupt the mechanism controlling mutually exclusive expression, implying that this phenomenon is independent of the expression of the antigenic protein PfEMP1, and therefore suggests that there are noncoding elements at each *var* locus that are essential and sufficient for this mechanism. The fact that we could get activation of endogenous *var* genes by removing drug pressure and subsequently recover complete silencing of the entire family by growing the parasites back in the presence of drug implies that mutually exclusive *var* expression in P. falciparum is a reversible, epigenetic process. In addition, all *var* genes, regardless of promoter type or chromosomal location, appear to be coordinately regulated in these transgenic parasites, indicating that all *var* genes must share similar intrinsic regulatory properties.

Previous work demonstrated that a transfected *var* promoter that is impaired in silencing and therefore constitutively active is not “counted” by the mechanism that controls mutually exclusive *var* gene expression [17]. The fact that the recombinant promoters described here that can be silenced are also “counted” implies that the mechanisms that control gene silencing and allelic exclusion may be linked. This hypothesis is supported by recent descriptions of two other *var* promoters that are constitutively active but not recognized by the mechanism that controls allelic exclusion. The first is the *var1csa* gene (also called *var*_common_). This gene appears to be impaired in silencing and is constitutively transcribed in many parasite isolates [24,25]. However, transcription of this gene does not affect expression of other *var* genes and it is therefore not recognized as part of the *var* gene family. The second example is from a transgenic line in which the *var2csa* gene has been disrupted [26]. This integration event also disrupted silencing of the gene and rendered the promoter constitutively active. Other *var* genes are also transcribed in this parasite line, indicating that the promoter of the disrupted gene is not recognized by the rest of the *var* gene family.

Silencing and activation of *var* promoters as well as recognition by the mechanism that controls mutually exclusive expression have now all been achieved in an episomal context. This indicates that all of these functions are encoded by the regulatory regions included in the plasmid constructs and that they are not dependent on characteristics of the chromosomal environment in which *var* genes reside. Thus specific chromosomal characteristics and elements that are frequently found in close proximity to *var* genes, for instance rep20 repeats or subtelomeric specific heterochromatin, are not likely to play a role in regulating *var* gene expression. Rather it seems likely that each individual *var* gene contains all of the elements that necessary for proper regulation and that these elements can be isolated within a plasmid construct. This hypothesis is consistent with the observation that two adjacent *var* genes that occupy the same chromosomal environment can assume different states of transcriptional activity.

The transgenic parasite lines described here provide an excellent tool for investigations into cytoadherence and virulence as well as the mechanism responsible for mutually exclusive *var* gene expression. For example, preliminary examination suggests that the switching rate may vary in the different transgenic cultures. In particular, the rate at which additional *var* genes are activated appears to be much slower in the parasite line containing the integration at the internal *var* gene containing the UpsC type promoter (compare Figures 4C and 5C). This may indicate that different *var* promoters have different intrinsic “on” and “off” switching rates, as was previously proposed [27], or alternatively that the apparent “on” rate of other *var* genes actually reflects a higher deletion rate of the transgene in this recombinant line. Future extensive cloning and expression analysis will directly address this question.

The ability to manipulate expression within the *var* gene family should allow studies into switching frequencies and the possibilities for either random or programmed switching between individual *var* genes. In addition, without PfEMP1 on the cell surface, the role of other red cell surface proteins in cytoadherence and antigenicity can now be more easily assessed. In particular, the roles of putative surface proteins including those encoded by *rif* and *stevor* genes, can be more definitively addressed.

## Materials and Methods

### Parasite culture and transfection.

All experiments utilized the P. falciparum NF54 line cultivated at 5% hematocrit in RPMI 1640 medium, 0.5% Albumax II (Invitrogen, Carlsbad, California, United States), 0.25% sodium bicarbonate, and 0.1 mg/ml gentamicin. Parasites were incubated at 37 °C in an atmosphere of 5% oxygen, 5% carbon dioxide, and 90% nitrogen. Parasites were transfected by using “DNA loaded” red blood cells as previously described [22]. Briefly, 0.2 cm electroporation cuvettes were loaded with 0.175 ml of erythrocytes and 50 μg of plasmid DNA in incomplete cytomix solution. Parasites were initially cultured in media containing 40 ng/ml pyrimethamine to select for stable episomes, followed by culturing in the presence of 20 μg/ml Blasiticidin S HCl (Invitrogen) to select for integration at the targeted *var* locus. Plasmid rescue experiments were performed by transforming E. coli competent cells with 500 ng of purified P. falciparum genomic DNA.

Clonal cultures originating from a single parasite were created by limiting dilution using 96-well microtiter plates as previously described [28]. Individual plates were screened for parasites during media changes on days 21, 25, and 30. Individual parasite cultures were then expanded to 20-ml cultures and used for DNA and RNA extraction.

### DNA constructs.

The plasmid pVLH/IDH [29] was previously described. This construct was used as a template for cloning. We amplified Aspergillus terreus blasticidin resistant gene from the plasmid pCBM-BSD [30], acquired from the American Type Culture Collection (Manassas, Virginia, United States) using the primers 5′-GC**GTTAAC**ATGCCTTTGTCTCAAGAAGAATCCACCCTC 3′ and 5′-GACGGG**AAGCTT**TGCTCCTCGGCCACGAAGTGC-3′ and cloned into pVLH/IDH in place of luciferase. *P. bergii hsp86* 3′ UTR was amplified from pHTK [31] using 5′-CCC**AAGCTT**GGATATGGCAGCTTAATG-3′ and 5′-CGC**GGATCC**CTACCCTGAAGAAGAAAA-3′ and used as a transcriptional terminator for the *bsd* gene to create the plasmid pVBB/IDH. Truncated *var* UpsC and UpsB upstream regions were amplified using the primers 5′-GG**GGTACC**GAAACATGTATGTTTTTATATGTATGT-3′ and 5′-GC**GTTAAC**TTTTGTTTATCGTTCGTGACTACATTATGTC - 3' (UpsB) and 5' - GG**GGTACC**TGCTATATTTAATTTTTTTTAAAAAA-3′ and 5′-GC**GTTAAC**ATAGTCTACCATTATAACATAAATAC-3′ (UpsC). The two truncated promoters were then cloned separately into the plasmid pVBB/IDH in place of the *var7b* promoter to create pV_b_BH/IDH and pV_c_BH/IDH.

### Southern blots and diagnostic PCR.

Analysis of the integrated constructs was performed using either Southern blots or diagnostic PCR across integration sites followed by sequencing. Southern blots were performed according to established protocols [32]. Briefly, genomic DNA isolated from recombinant parasites was digested to completion by restriction enzymes and subjected to gel electrophoresis using 1% agarose in Tris/Borate/EDTA (TBE). The DNA was transferred to high-bond nitrocellulose membrane by capillary action after alkaline denaturation. DNA detection was performed using the Amersham nonradioactive detection kit and the manufacturer's protocols.

### Genomic DNA extraction.

Infected RBCs were pelleted by centrifugation at 6000 rpm. After discarding the supernatant, the pellet was divided into two microcentrifuge tubes followed by resuspension in 500-μl phosphate-buffered Saline and 20-μl 10% Saponin. Parasites were pelleted by centrifugation and washed twice with 1000 μl PBS. The parasite pellet was then taken up in 200-μl TSE buffer (100 mM NaCl, 50 mM EDTA, 20 mM Tris, [pH 8]) to which 40 μl of 10% SDS and 20 μl 6M NaClO_4_ were added. This suspension was placed on a rocker overnight and the DNA extracted with phenol/chloroform the next morning. The DNA was precipitated from the final aqueous phase with ethanol and resuspended in 10 μl sterile dH_2_O. Final DNA concentration was verified by absorbance at 260 nm.

### RNA extraction and realtime RT-PCR for assaying expression of the *var* gene family.

RNA was extracted from synchronized ring stage parasites 16–18 h post-invasion. RNA extraction was performed with the TRIZOL LS Reagent (Invitrogen) as previously described [33]. RNA to be used for cDNA synthesis was purified on PureLink column (Invitrogen) according to manufacturer's protocol. Isolated RNA was then treated with Deoxyribonuclease I (Invitrogen) to degrade contaminating gDNA. cDNA synthesis was performed with Superscript II Rnase H reverse transcriptase (Invitrogen) with random primers (Invitrogen) as described by the manufacturer. cDNA was synthesized from 800 ng total RNA in a reaction volume of 40 μl. For each cDNA synthesis reaction, a control reaction without reverse transcriptase was performed with identical amounts of template. For realtime quantitative RT-PCR reactions to detect transcription from all *var* genes present in the 3D7 genome, we employed the primer set of Salanti et al. [23] with the following modifications. We added an additional primer pair for PF08_0107: 5′-CCTAAAAAGGACGCAGAAGG-3′ and 5′-CCAGCAACACTACCACCAGT-3′ and designed separate primer sets for PFD1005c: 5′-ACGATTGGTGGGAAACAAAT-3′ and 5′-CCCCATTCTTTTATCCATCG-3′ and for PFD1015c: 5′-AAAGGAATTGAGGGGGAAAT-3′ and 5′-TAAACCACGAAACGGACTGA-3′.

All reactions included the three control genes published by these authors: seryl-tRNA synthetase (PF07_0073), fructose biphsphate aldolase (PF14_0425), and actin (PFL2215w); however, we added another two control sets: arginyl-tRNA synthetase (PFL0900c) using 5′-AAGAGATGCATGTTGGTC-3′ and 5′-GTACCCCAATCACCTACA-3′ and glutaminyl-tRNA synthetase (PF13_0170) using 5′-GGCACTTCAAGGGTACCT-3′ and 5′-TAATATAGCCTCACAAGC-3′.

To quantify *bsd* transcription levels we used the primers 5′-TTGTCTCAAGAAGAATCCAC-3′ and 5′-TCCCCCAGTAAAATGATATAC-3′. Amplification efficiency was verified by performing amplifications using different concentrations of genomic DNA as templates. Reactions were performed at a final primer concentration of 0.5 μM using Biorad ITAQ SYBR green Supermix in 20-μl reactions on an ABI Prism 7900HT. All runs were done in triplicate and yielded virtually identical Ct (cycle threshold) values. The Δ Ct for each individual primer pair was determined by substracting the measured Ct value from the Ct value of the control seryl-tRNA synthetase (User bulletin 2, Applied Biosystems, http://www.appliedbiosystems.com). Δ Cts were then converted to relative copy numbers with the formula 2^ Δ Ct^.

### Western blot analysis.

Late-stage parasites (trophozoite and schizonts) were isolated from 2 × 10^8^ infected erythrocytes by percol/sorbitol gradient centrifugation, washed in PBS buffer, and the erythrocytes lysed in 500 μl NET/1% TX-100 (150 mM NaCl, 5 mM EDTA, 50 mM Tris, [pH 8]), supplemented with protease inhibitor cocktail tablets (Roche, Basel, Switzerland). Released parasites were collected by centrifugation (14,000 g, 5 min, 4 °C), washed in the same buffer, and disrupted in 200 μl TSA (20 mM Tris [pH 8], 150 mM NaCl and 0.02% sodium azide) using a Gilson pipette. 50 μl 10% SDS was added and parasite DNA sheared by repeated passage through a needle. Triton insoluble and SDS soluble fractions were isolated and solubilized in SDS-loading buffer. Proteins were subjected to SDS-PAGE (10% polyacrylamide) and electroblotted to PVDF membrane. Immunodetection was carried out using an alkaline phosphatase–conjugated secondary antibody (Kirkegaard & Perry Laboratories, Gaithersburg, Maryland, United States) and developed in NBT/BCIP solution (Sigma-Aldrich, St. Louis, Missouri, United States) for 5–15 min.

### Antisera.

Immuno-detection of PfEMP1 was carried out using a polyclonal rabbit antibody to the conserved cytoplasmic domain (α ATS) of PfEMP1 (T. Fagan and C. I. Newbold, unpublished data). As a control for loading, we used mouse sera recognizing the abundant P. falciparum endoplasmic reticulum protein, *Pf*39 [34,35].

## Abbreviations

Ct: cycle threshold
PCR: polymerase chain reaction
